# Bogus Diplomas

**Published:** 1877-03

**Authors:** 


Bogus Diplomas.—The following correspondence will explain itself:

[Copy.]

Camp Stambaugh, Wyoming Ter., Feb. 7, 1877.

*Surgeon-General U. S. Army, Washington, D. C.:*

Sir:—I have the honor to enclose herewith, for the information of the Surgeon-General U. S. A., a communication received by me on the 6th inst.

Very respectfully your obedient servant,
[Signed] Charles H. Dodge,
*Hospital Steward U. S. A.*

[Copy.] Buffalo, N. Y., January, 1877.

Sir:—Do you want a medical diploma? Some hospital stewards have obtained diplomas through our agency, and are practicing successfully. Many are well qualified, but lack diplomas. The agent, having been a hospital steward during the late war, knows whereof he writes. Fully one-fourth of the physicians in the United States have procured their diplomas through our influence.

Through the private agent of a medical college which cannot be questioned, you are proffered a diploma for the consideration of fifty ($50.00) dollars. *No security is given, but the diploma will positively be forwarded by registered mail.* Send money by registered, or ordinary letter, *at the agent's risk.* A limited number only will be disposed of. If you cannot invest, please hand this to one who may.

An early reply is particularly requested.

Yours truly,

JOSEPH P. DONNER.

Address 580 Louisiana street, Buffalo, N. Y.

[We have received several copies of the above advertisement, forwarded to us by hospital stewards stationed in every section of the country. There is no doubt that the agent is making strenuous efforts to extend his business. Cannot some of our friends in Buffalo look after him ?—ED.] *Med. Record.*

We have also heard of this diploma dealer. Hospital stewards who are willing to receive diplomas in that way better by all means send him fifty dollars. When they get the diploma we wish they would send us word, or a copy.

